# Grape seed extracts modify the outcome of oxaliplatin in colon cancer cells by interfering with cellular mechanisms of drug cytotoxicity

**DOI:** 10.18632/oncotarget.15139

**Published:** 2017-02-07

**Authors:** Letizia Porcelli, Rosa Maria Iacobazzi, Anna Elisa Quatrale, Carlo Bergamini, Nunzio Denora, Pasquale Crupi, Donato Antonacci, Anita Mangia, Giovanni Simone, Nicola Silvestris, Amalia Azzariti

**Affiliations:** ^1^ Experimental Pharmacology Laboratory, IRCCS Istituto Tumori Giovanni Paolo II, Bari, Italy; ^2^ CRA-UTV Research Unit for Viticulture and Enology in Southern Italy, Turi, Italy; ^3^ Department of Pharmacy-Drug Sciences, University of Bari Aldo Moro, Bari, Italy; ^4^ Biomorphology Laboratory, IRCCS Istituto Tumori Giovanni Paolo II, Bari, Italy; ^5^ Pathological Anatomy, IRCCS Istituto Tumori Giovanni Paolo II, Bari, Italy; ^6^ Medical Oncology, IRCCS Istituto Tumori Giovanni Paolo II, Bari, Italy

**Keywords:** grape seed extracts, colon cancer cells, oxaliplatin, apoptosis, transport systems

## Abstract

Grape seed extracts are commonly utilized as dietary supplements for their antioxidant properties, even from cancer patients. However, whether these natural extracts interfere with chemotherapeutics utilized in colon cancer treatment is still poorly investigated. The cytotoxicity of extracts from Italia and Palieri cultivars either alone or in combination with oxaliplatin was evaluated in colon cancer cells. Grape seed extracts displayed anti-proliferative activity depending on the concentration utilized through apoptosis induction. In combination, they affected the activation of Erk1/2 and counteracted the intrinsic and the extrinsic pathway of apoptosis, the DNA damage and the generation of ROS induced by oxaliplatin. Noteworthy grape seed extracts strongly enhanced the uptake of oxaliplatin into all cells, by affecting the cell transport system of platinum. The addition of these natural extracts to oxaliplatin strongly reduced the cellular response to oxaliplatin and allowed a huge accumulation of platinum into cells. Here, we shed light on the chemical biology underlying the combination of grape seed extracts and oxaliplatin, demonstrating that they might be detrimental to oxaliplatin effectiveness in colon cancer therapy.

## INTRODUCTION

Advances in chemotherapy have contributed to the increased survival rates of cancer patients. However, the clinical efficacy of chemotherapy is often limited by its non-cancer specific effects causing damage to proliferating normal cells, especially those of the bone marrow and of the gastrointestinal tract. Besides these primary side effects, there are a number of other toxicities that the patient has to bear during chemotherapy such as fatigue, nausea, vomiting, diarrhea, etc. These are often approached by administering to patients supplements to improve their general status [1, 2].

Moreover, to minimize these adverse effects and perhaps even be able to increase the effectiveness of chemotherapy, a large part of the scientific community has focused on the study of natural plant products and their interactions with therapy. Today, bioactive phytochemicals, particularly those already present in the diet, offer promising options for the development of more effective strategies for the prevention or treatment of cancers and they are often utilized as complementary or alternative medicine [3–8].

An example are proanthocyanidins found in grape seeds as dimers, trimers, and other oligomers of flavan-3-ols. Due to their natural ROS modulating and natural free radical scavenging abilities, grape seed extract (GSE) is consider a dietary health supplement [9, 10]. GSEs have demonstrated chemopreventive and/or chemotherapeutic effects in various cancer cell cultures and animal models, evidencing that they have not only anti-inflammatory properties, but also therapeutic activity in skin, oral, colorectal, prostate, breast, lung, and gastric cancers [9].

However, when GSE was studied in combination with chemotherapy on the viability of colon cancer cells in order to enhance chemotherapeutics’ action without compromising the well-being of patients, different effects of the GSE components were reported. Indeed, a number of studies has shown how the degree of polymerization and galloylation of the GSE procyanidins is the main responsible for their antiproliferative activity on cancer cells [11, 12].

Moreover, Cheah and coauthors have found that grape seed extract (GSE) has not only the ability to kill cancer cells but also made more powerful 5-fluorouracil (5-FU) at the tested concentration, opening the possibility to utilize these natural extracts as enhancers of chemotherapy [13].

Nevertheless, because the analytical composition of GSE from different cultivars differs greatly in flavanols content, an important question can rise whether different GSE samples have comparable activity and trigger the same molecular mechanisms on a given biological system. Therefore, our study starts from the need to expand the analysis of the ability of GSE to modulate also the other chemotherapeutic drugs which represent the currently accepted standard of care (oxaliplatin, 5-FU and irinotecan) for colon cancer patients. In this context, we investigated the effects of the natural extracts obtained from Italia and Palieri grape seeds (I-GSE and P-GSE), of which we have already demonstrated the capability to trigger apoptosis in Caco-2 cells [14], either alone and in combination with oxaliplatin, in primary-derived HT-29 and HCT116 and metastatic Colo205 and Lovo colon cancer cell lines, representative of this pathology.

This study provides insights into the mechanism by which GSEs induce apoptosis in these cells and a convincing rationale of their pharmacological activity against colon cancer cells and points out that, when GSEs are administered with oxaliplatin, a strong interplay takes place in their mechanisms of action suggesting that the association of GSEs with oxaliplatin-based chemotherapy requires many precautions.

## RESULTS

To explore the relevance of grape seed extracts on the response to chemotherapy in the anti-colon cancer treatment, GSEs were obtained from two different cultivars *Italia* (I-GSE) and *Palieri* grape (P-GSE) and characterized by HPLC-MS/MS analyses. As reported in Table 1, 25 flavan-3-ols were identified in both the extracts and namely distinguishable, on the basis of their common [M-H]- and fragmentation MS/MS patterns, into 2 catechins, 6 procyanidin B-type dimers, 2 procyanidin dimers gallate, 5 procyanidin trimers, 8 procyanidin trimers gallate, and 3 procyanidin tetramers, respectively. However, with regard to the relative amount of these compounds, the difference between Italia and Palieri GSE was mainly related to the percentage of catechins, procyanidins dimers and trimers (Table 1).

**Table 1:** HPLC-MS/MS characterization of flavan-3-ols present in the grape seed extracts (cv. Italia and M. Palieri)

Flavan-3-ols	RT (min)	[M-H]^-^	MS/MS	cv. Italia (%)	cv. M.Palieri(%)
Procyanidin trimer-1	6.1	865	695(100),577(40.0),451(18.4),407(24.8)		
Procyanidin tetramer-1	13.5	1153	1027(83.0),983(90.5),865(100),577(89.7)		
Procyanidin B1	14.4	577	451(93.0),425(76.3),407(100),289(77.0)		
Procyanidin B3	14.9	577	451(95.0),425(83.7),407(100),289(62.2)		
Procyanidin trimer-2	15.7	865	695(100),577(81.2),451(29.5),407(50.2)		
Procyanidin trimer gallate-1	15.9	1017	865(35.7),729(100),695(68.8),577(48,3)		
Catechin	16.0	289	289(100),245(43.3),205(8.9)		
Procyanidin trimer-3	17.1	865	695(100),577(67.4),451(18.9),407(29.9)		
Procyanidin tetramer-2	17.3	1153	1027(53.0),983(75.9),865(100),577(65.5)		
Procyanidin B2	17.7	577	451(64.7),425(100),407(83.6),289(47.9)		
Procyanidin B6	18.5	577	451(21.6),425(100),407(49.6),289(20.4)		
Procyanidin trimer-4	18.9	865	695(100),577(78.4),451(30.7),407(52.4)		
Procyanidin trimer gallate-2	19.5	1017	865(25.7),729(100),695(28.8),577(28,3)		
Procyanidin trimer gallate-3	19.9	1017	865(100),727(49.7),695(45.7),577(28,3)		
Epicatechin	20.3	289	289(100),245(33.1),205(8.7)		
Procyanidin gallate-1	21.6	729	577(100),451(50.8),407(81.8),289(21.5)		
Procyanidin trimer gallate-4	21.9	1017	865(37.7),729(87.8),695(19.6),577(33.0)		
Procyanidin gallate-2	22.9	729	577(96.2),451(82.0),407(100),289(56.1)		
Procyanidin trimer gallate-5	23.0	1017	865(63.0),729(100),695(25.8),577(21.1)		
Procyanidin trimer-5	23.5	865	695(100),577(71.3),451(31.7),407(42.4)		
Procyanidin tetramer-3	25.4	1153	1027(57.1),983(66.9),865(83.1),577(49.1)		
Procyanidin trimer gallate-6	26.3	1017	865(44.1),729(100),695(37.5),577(36.7)		
Epicatechin gallate	33.4	441	331(95.8),289(100),245(13.1)		
Procyanidin trimer gallate-6	35.5	1017	865(24.9),729(100),695(38.7),577(15.3)		
Procyanidin trimer gallate-7	36.3	1017	865(32.4),729(100),695(15.9),577(14.9)		
Catechins				31.88	43.46
Catechins gallate				8.05	7.05
Procyanidin dimers				50.12	42.80
Procyanidin dimers gallate				3.79	3.15
Procyanidin trimers				5.17	2.78
Procyanidin trimers gallate				0.81	0.63
Procyanidin tetramers				0.17	0.13

[M-H]^-^: deprotonated molecule; MS/MS: product ions.

Therefore, the evaluation of the efficacy of GSEs as anticancer agents was performed in colon cancer cell lines together with the investigation of the capability of GSEs to affect oxaliplatin effectiveness which represents a gold standard in the treatment of this pathology.

### GSEs alone

**Figure 1:**
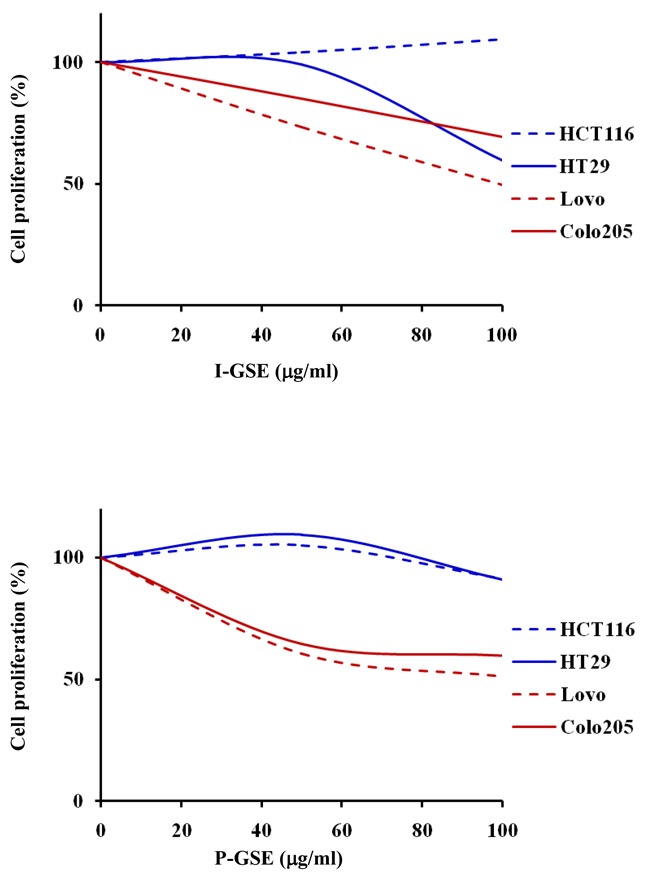
Dose-effect plots showing the inhibition of cell proliferation by GSEs HCT116, Colo205, LoVo and HT-29 cells were incubated for 3 days with 0.1-100 ug/ml I-GSE or P-GSE, then the survival of cells was determined using the MTT assay and expressed as percentage of untreated cells. Each experiment was repeated at least three times.

To investigate whether the behavior of colon cancer cells in the presence of the natural extracts depends from the tumor origin, the panel of cell lines included cells from primary tumors (HCT116 and HT-29) and from metastatic sites (LoVo and Colo205). The exposure to I-GSE and P-GSE, ranging between 0.1 ug/ml and 100 ug/ml, for 3 days reduced cell proliferation differently in the two models, probably because of their different composition and with an higher efficacy in the metastatic cells (Figure 1) with the exception of HT-29 which at high dose of I-GSE were inhibited of about 50%. The following characterizations on the efficacy of the combined administration of GSE plus oxaliplatin were performed using both grape seed extracts at 50 and 100 ug/ml, two concentrations that should put in evidence the modulation of the response to chemotherapy because yet slight active alone and much lower than the doses of GSEs administered as food supplements which are approximately 200mg/die. The capability of GSEs to perturb cell cycle progression was investigated by FCM analysis showing only a slight capability (about 10%) of these natural extracts to selectively block cells in G0/G1phase (data not showed).

### Combined approach

**Figure 2:**
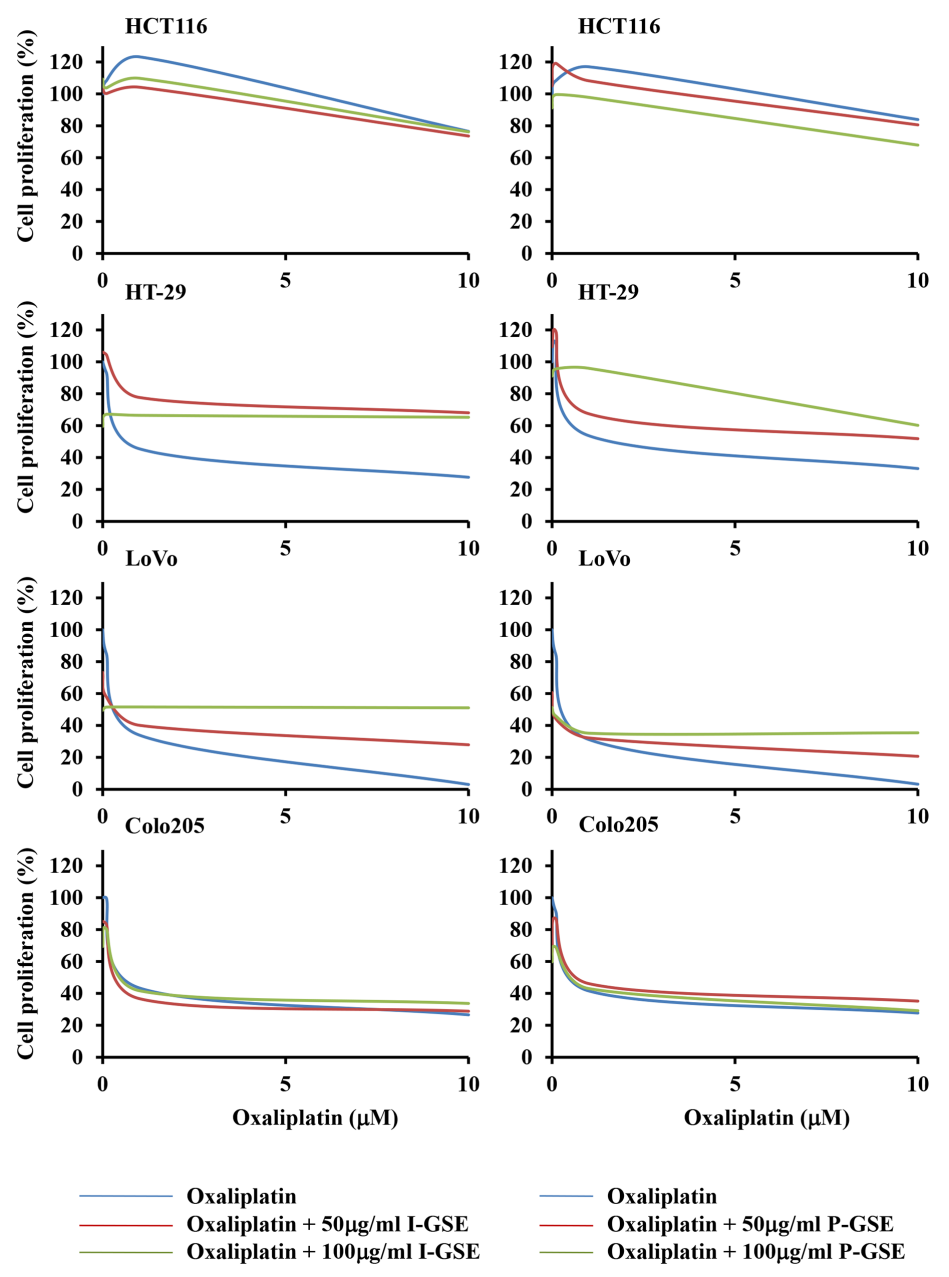
Dose-effect plots showing the inhibition of cell proliferation by oxaliplatin plus GSEs HCT116, Colo205, LoVo and HT-29 cells were incubated for 3 days with 0.1 – 1 – 10 uM oxaliplatin or in combination with 50 and 100 ug/ml of I-GSE and P-GSE. The survival of cells was determined using the MTT assay and expressed as percentage of untreated cells. Each experiment was repeated at least three times.

Oxaliplatin is usually included in quite all standard strategies utilized against colon cancer and all cells, utilized in this study, were screened for their sensitivity to this platinum-derivative (IC50s, after 3 days drug-exposure, are 32±3.6, 2.57±0.89, 0.58±0.13 and 2.6±0.52uM for HCT116, HT-29, LoVo and Colo205, respectively). In the combination study, oxaliplatin was utilized at three scalar concentrations 0.1, 1 and 10uM, taking into account that the maximal blood concentration of this drug varies between and 1.39 and 3.39ug/ml (4.8 and 8.5 uM) [15, 16]. The simultaneous exposure to GSE and oxaliplatin showed a different response of colon cancer cells independently from the tumor origin and from the sensitivity to the chemotherapeutic agent; indeed, HCT116 and Colo205, also taking into account their different sensitivity to oxaliplatin, showed a lack of modulation after the addition of GSEs while the other two cell lines, HT-29 and Lovo, were markedly protected from the oxaliplatin-induced cytotoxicity. In Lovo cells the extent of the rescue was dependent on the concentration utilized of both Italia and Palieri-GSEs, while in HT-29 a dose-dependent protective effect against oxaliplatin was observed only with the P-GSE and did not with I-GSE (Figure 2). The analysis of cell cycle perturbation when cells were exposed to GSE plus oxaliplatin evidenced, as known [17], a slight increase in G2/M phase after the platinum derivative exposure while no modification were present after the addition of GSEs in all cell lines (data not showed).

The absence of correlation between cell response to GSE plus oxaliplatin and either tumor origin and basal sensitivity to this platinum derivative suggested to investigate if the natural extracts reduced the cytotoxicity of the drug affecting selective mechanisms of response to oxaliplatin. However, due to the aforementioned similar action of the two extracts, only results of I-GSE are showed when are similar to those obtained with P-GSE. Moreover, the kinetic results obtained by combining GSEs and oxaliplatin highlighted two different general response of the colon cancer models included in our study allowing to discriminate HCT116 and Colo205 as the cellular models in which the addition of both GSEs did not affect the cytotoxicity of oxaliplatin and HT-29 and LoVo in which the addition of both GSEs reduced the response to the drug.

### LoVo and HT-29 cells: GSEs reduced apoptosis induction by oxaliplatin

**Figure 3:**
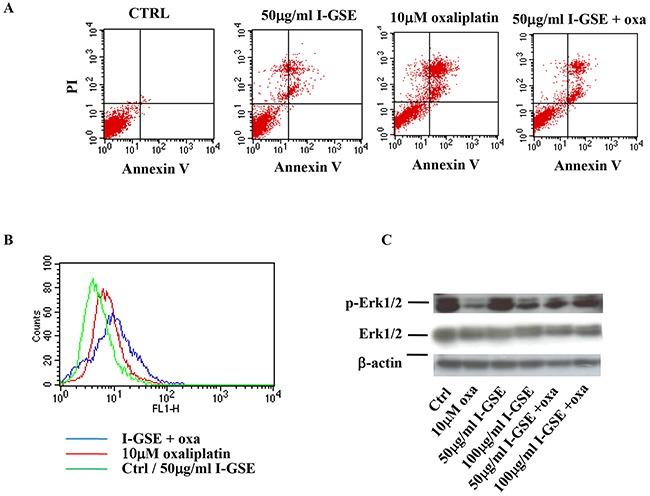
HT-29 response to oxaliplatin and/or I-GSE HT-29 cells were incubated with oxaliplatin and/or I-GSE for 1 or 3 days. Apoptosis induction, after 3 days of drug(s)’ exposure, was determined with Annexin V/PI assay **A.** the modulation of γH2AX, after 1 day of drug(s)’ exposure, by FCM **B.** and p-Erk1/2/Erk1/2, after 3 days of drug(s)’ exposure, by Western Blotting **C.**

In Figure 3, results on HT-29 cells are reported and are similar to those obtained in Lovo cells. HT-29 are “protected” by GSEs after oxaliplatin administration with a marked recovery of cell proliferation as respect to cells treated with the chemotherapeutic agent alone (See Figure 2). The apoptosis determination confirmed the kinetic data. As reported in panel A of Figure 3 in which the AnnexinV/PI staining of untreated and treated cells are showed, the apoptosis is much more activated by oxaliplatin than by the GSEs. Their combination resulted in an apoptosis induction similar to that induced by the GSEs alone, strongly suggesting a blockage of oxaliplatin apoptogenic activity.

### LoVo and HT-29 cells: how can GSEs have such activity?

Oxaliplatin induces DNA damage that cells try to repair by initiating the complex signaling of DNA damage response (DDR) [18] with the phosphorylation of the histone variant H2AX on Ser139 (γ-H2AX), which forms nuclear foci at sites of DNA damage. In order to evaluate the effects of GSEs on the activation of the DDR, the concentration of γH2AX was determined in response to treatment with oxaliplatin, with GSE and with the combination oxaliplatin plus I-GSE. As expected, oxaliplatin increased the γH2AX expression, evidenced by the right shift of the FL-1H fluorescence (red line); the I-GSE did not increase the formation of γ-H2AX foci, however when I-GSE was added to oxaliplatin their formation increased, as evidenced by the additional right shift of the FL-1H fluorescence (blue line) (Figure 3B); suggesting that the combined therapy increased the signal of DNA damage and perhaps triggered a mechanism leading to the activation of DDR with a consequent cellular recovery from the DNA-targeted drug. The analysis of p-Erk1/2 expression, which is known to be affected by oxaliplatin, showed that this drug strongly reduced the phosphorylation of Erk1/2 according to the inhibition of cell proliferation. I-GSE seemed to have not a direct activity on this effector at low doses, but, when given together with the chemotherapeutic agent, it induced the recovery of Erk1/2 phosphorylation, which was the stronger the higher the concentration of I-GSE, compared to oxaliplatin treated sample (Figure 3C). All these results suggested that in LoVo and HT-29 cells, GSEs reduced the cytotoxic response to oxaliplatin.

### Colo205 and HCT116 cells: GSEs didn't modify apoptosis induction by oxaliplatin, what happens to the phosphorylation of H2AX and Erk1/2?

**Figure 4:**
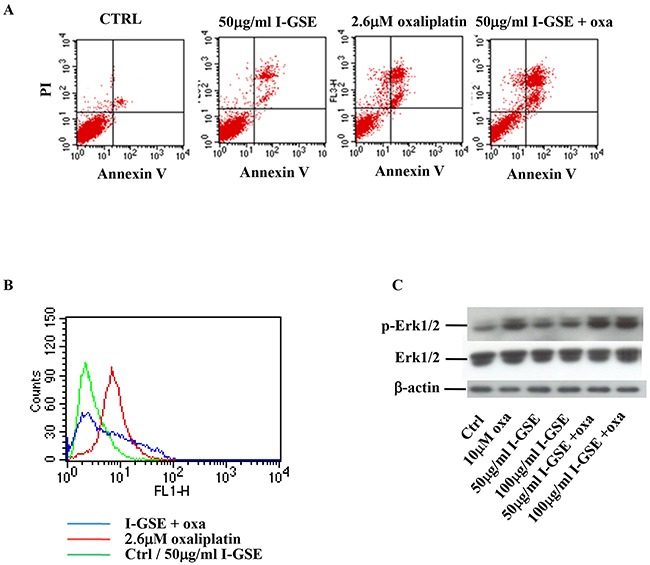
Colo205 response to oxaliplatin and/or I-GSE Colo205 cells were incubated with oxaliplatin and/or I-GSE for 1 or 3 days. Apoptosis induction, after 3 days of drug(s)’ exposure, was determined with Annexin V/PI assay **A.** the modulation of γH2AX, after 1 day of drug(s)’ exposure, by CFM **B.** and p-Erk1/2/Erk1/2, after 3 days of drug(s)’ exposure, by Western Blotting **C.**

In the other colon cancer models (Colo205 and HCT116), the apoptosis analysis confirmed the kinetic results, showing that the addition of GSEs didn't affect the cytotoxicity of oxaliplatin, as demonstrated by the persistent Annexin V/PI positive cells population (upper right quadrant), comparable to that of oxaliplatin given alone (Figure 4A). To evaluate the effects elicited by GSEs on oxaliplatin-induced DNA damage and Erk1/2 activation even in Colo205 and HCT116 the amount of H2AX and Erk1/2 phosphorylation were analyzed. As reported in the output file of the analysis performed on Colo205 cells, the histone 2AX is phosphorylated by the platinum derivative, but the addition of I-GSE strongly reduced its expression suggesting that the natural extracts reduced the DNA damage signal (Figure 4B). Regarding oxaliplatin ability to affect p-Erk1/2 expression, in these cells the results were opposite to HT-29 and Lovo cells; i.e., 1 day treatment with oxaliplatin increased the phosphorylation (data not showed) and this effect was persistent after three days of continuous exposure (Figure 4C). The addition of GSEs alone at both concentrations slightly increased p-Erk1/2, but when given in combination with oxaliplatin they failed to counteract oxaliplatin-induced activation of Erk1/2. The activation of Erk1/2 by oxaliplatin might be consistent with data reported in literature [19] which regarded the persistent p-Erk1/2 increase as responsible for apoptosis induction; thus perhaps p-Erk plays a crucial role in the apoptotic effects of oxaliplatin in Colo205 and HCT116, which is not affected by the addition of GSEs.

### Apoptosis mechanisms affected by GSEs

Previous results on the opposite effects elicited by oxaliplatin and by the combination of GSEs plus oxaliplatin on Erk1/2 activity and on the induction of apoptosis, in LoVo and HT-29 vs Colo205 and HCT116, suggested to further study the mechanisms of cell death by analyzing the involvement of the intrinsic and extrinsic pathways of apoptosis. The characterization was carried out in Colo205 and HT-29.

**Figure 5:**
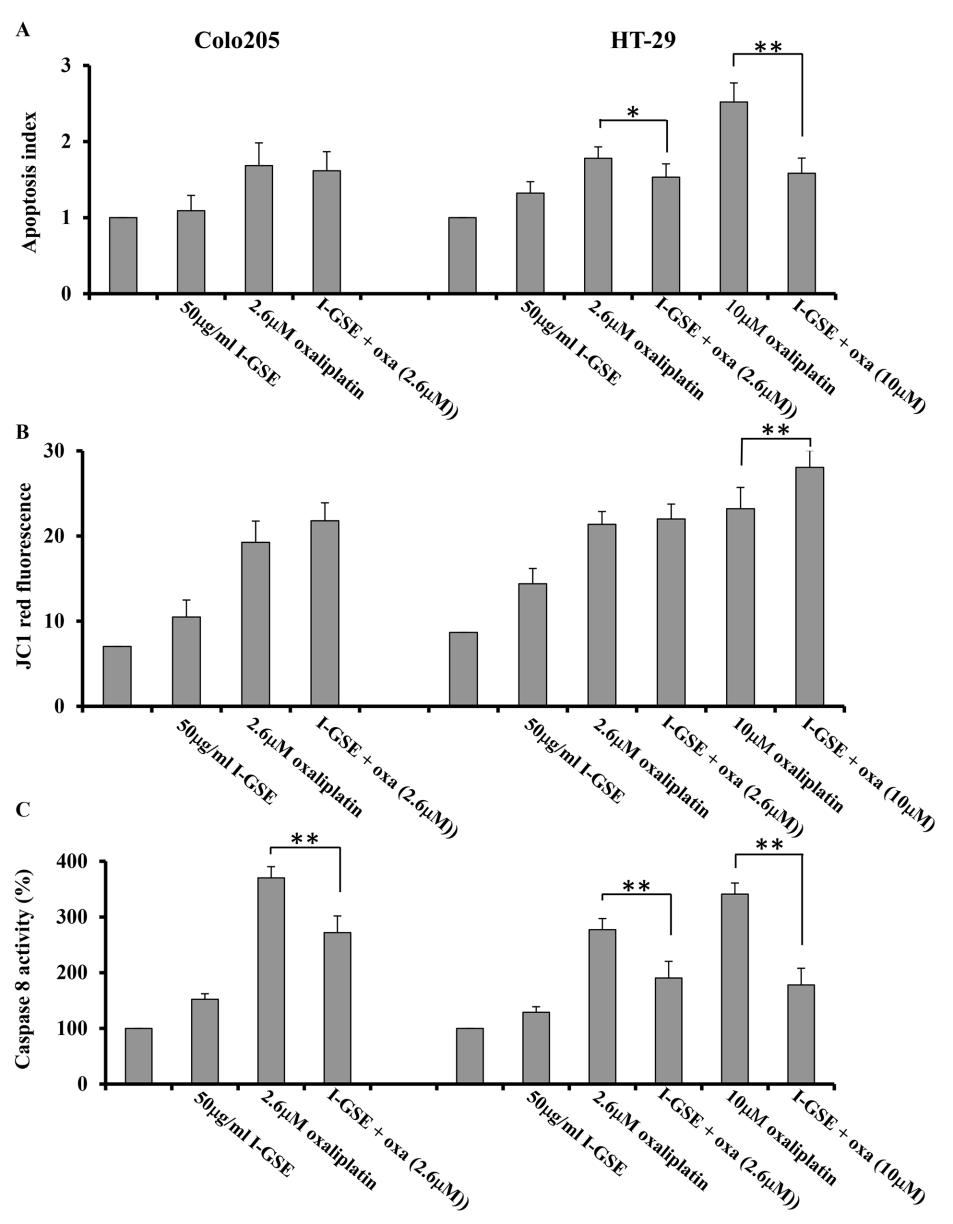
Mechanisms of apoptosis induction Colo205 and HT-29 cells were incubated with 50 ug/ml I-GSE, with oxaliplatin and the combination for 1 day, apoptosis induction was determined using the Cell Death Elisa Kit, and expressed as apoptotic index intreated cells compared to untreated ones. Shown are the data ±SD, relative to the results from at least three independent experiments **A.** the mitochondrial membrane potential for the determination of mitochondrial damage was assessed with the fluorescent dye JC-1 and analyzed by FCM. Shown are the data of the quantification of red fluorescence, correspondent to the reduction of mitochondrial membrane potential from the measurements of at least three independent experiments **B.** the quantification of caspase-8 activation was through the Caspase 8 Colorimetric Activity Assay Kit. The data are expressed as percentage of untreated cells ±SD of the results from at least three independent experiments **C.** The significance of results are:*<0.05; **< 0.001.

The activation of apoptosis was measured by the DNA laddering formation assay too and data are reported as apoptosis index. The results are similar to those already showed in Figures 3 and 4 obtained by AnnexinV/PI staining assay. In fact, in Colo205 cells the addition of I-GSE to oxaliplatin didn't modify its capability to induce apoptosis in both assays and in HT-29 cells, the apoptosis index resulted to be reduced of about 62% (from 2.52 to 1.58) when I-GSE was added to 10μM oxaliplatin (Figure 5A) and a similar reduction, from 20.39% to 10.98% of late apoptosis (in the right upper quadrant of Figure 3A) resulted by AnnexinV/PI staining assay. I-GSE induced a no significant increase of apoptosis. Oxaliplatin, at the IC50 doses, killed cells with the same efficiency in both models while the combination with GSE did not modify apoptosis in Colo205 and did it in HT-29. The reduction of apoptosis in HT-29 was much more evident when I-GSE was added to 10μM oxaliplatin, in agreement to kinetic results (Figure 5A). The data obtained with I-GSE are also representative of those with P-GSE.

Focusing on the effect of I-GSE on the intrinsic pathway of apoptosis, the impairment of mitochondria was evaluated by measuring the oxaliplatin-induced mitochondrial depolarization with JC1 staining. As reported in Figure 5B, both the drug and the natural extracts depolarized mitochondria either in Colo205 and in HT-29, albeit the extent of mitochondria impairment was greater in the latter cell line. The combination of GSEs with oxaliplatin at IC50s did not result in a significant alteration of membrane potential in the two cellular models as respect the chemotherapeutic drug alone, while it was significantly increased when I-GSE was added to high dose-oxaliplatin in HT-29. These last results seemed in disagreement with the kinetic and apoptosis data prompting us to investigate the effects of I-GSE addition to oxaliplatin on the extrinsic pathway of apoptosis, involving the activation of death receptors. To this purpose the activation of caspase-8 was evaluated as a crucial event in the death-receptor-initiated apoptosis. In Figure 5C, the data on the activation of caspase-8 demonstrated that it occurred in both cell lines. The activation of caspase-8 by oxaliplatin was stronger in Colo205 than in HT-29 at IC50s, while in both cellular models I-GSE slightly increased caspase-8 activation and reduced the oxaliplatin-dependent caspase-8 activation of about 30%. Interestingly in HT-29, I-GSE reduced caspase-8 activation to a greater extent (50%) when given in combination to oxaliplatin at 10μM.

### Which is the mechanism engaged after pro-caspase-8 activation and responsible for apoptosis?

The level of caspase-8 might be sufficient to directly initiate the effector caspases cascade or the death receptor-initiated apoptosis might require amplification through the engagement of the mitochondrial intrinsic pathway by means of a cellular effector that behaves as a link. Substrates of caspase-8 are caspase-3 and/or the BH3-only protein BID of the Bcl-2 family. The first leads to the activation of effector caspases cascade, while the latter, once activated as t-BID (truncated form of BID), translocates to mitochondria where it induces permeabilization of the outer mitochondrial membrane through the pro-apoptotic proteins Bax and/or Bak, allowing the release of pro-apoptotic factors from mitochondria and triggering effector caspases activation and enabling that the death receptor-dependent apoptosis signaling and the mitochondrial pathway are interconnected [20].

**Figure 6:**
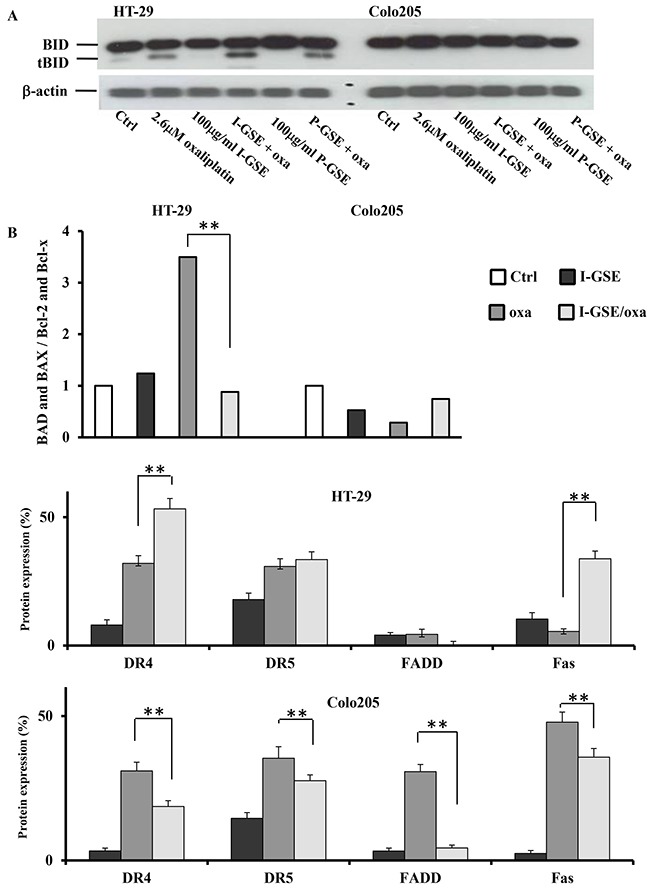
BID activation and proteins involved in apoptotic pathways BID cleavage determination in HT-29 and Colo205. Protein expression was determined by Western blotting on whole lysates from cells treated with oxaliplatin, GSEs and combined treatments after 3 days **A.** expression quantification of proteins from the densitometric analysis of the proteome profiler human apoptosis Array kit. The cells were treated as previously described for 3 days. Shown are the percentage of proteins expression in the treated samples respect to untreated cells ±SD, relative to two independent experiments **B.** The significance of results are: *<0.05; **< 0.001.

The formation of t-BID was evaluated by western blotting on HT-29 and Colo205 cells treated with GSE and with oxaliplatin or with the combination (Figure 6). Results evidenced that in HT-29 but not in Colo205 cells, the activation of the death-receptor-initiated apoptosis proceeded through the cleavage of Bid. In HT-29, oxaliplatin induced the cleavage of Bid while GSE did not; however, when they were combined, t-Bid increased consistently with the increased damage to the outer mitochondrial membrane, evidenced by JC-1 analysis after the combination in this cell line (Figure 5B).

### Analysis of proteins involved in apoptosis affected by GSEs

To further explore the mechanisms leading to caspase-8 activation in Colo205 and HT-29 cells, the Human Apoptosis Array kit was utilized. Only in HT-29, both GSEs and oxaliplatin increased DR4 and Fas levels which were much more expressed when the two drugs are combined conversely, the expression of DR5 seemed to be not modified by the addition of GSE to oxaliplatin. The analysis of the Fas-associated death domain protein (FADD), the adaptor protein which allows the formation of DISC (death-inducing signaling complex) triggering apoptosis, showed that GSE was able to completely counteract the expression of FADD induced by oxaliplatin in both models suggesting a reduction of death-receptors dependent apoptosis by I-GSE through the downregulation of FADD, which could account for the reduction of caspase-8 activation, induced by the combination, in both cell lines (see Figure 5C)

Focusing on the mitochondrial pathway of apoptosis it is strictly dependent by the permeabilization of the outer mitochondrial membrane by proapoptotic members of the Bcl family. The Bcl-2 family of proteins contains both anti-apoptotic proteins, such as Bcl-2 and Bcl-xl, and pro-apoptotic proteins, such as BAD and BAX. The analysis of such proteins was performed by means of the Human Apoptosis Array kit. As the balance between these molecules is crucial in determining sensitivity or resistance to mitochondrial apoptosis, the ratio of the expression of the pro-apoptotic proteins versus the anti-apoptotic ones has been evaluated in HT-29 and Colo205 after treatments. As depicted in the Figure 6, the fold change of the ratio strongly increased after treatment with oxaliplatin in HT-29; however a drastic reduction of the ratio was observed when GSE was added; this because the anti-apoptotic proteins expression (data not showed) elicited by I-GSE, shifted the balance towards antiapoptotic effects, thus attenuating apoptosis induction by mitochondria.

Contrariwise in Colo205, the ratio was quite similar after each treatment, and no significant modulation was found in the combination, suggesting the absence of the Bcl-2 family of proteins in determining cells susceptibility to the mitochondrial apoptosis.

**Figure 7:**
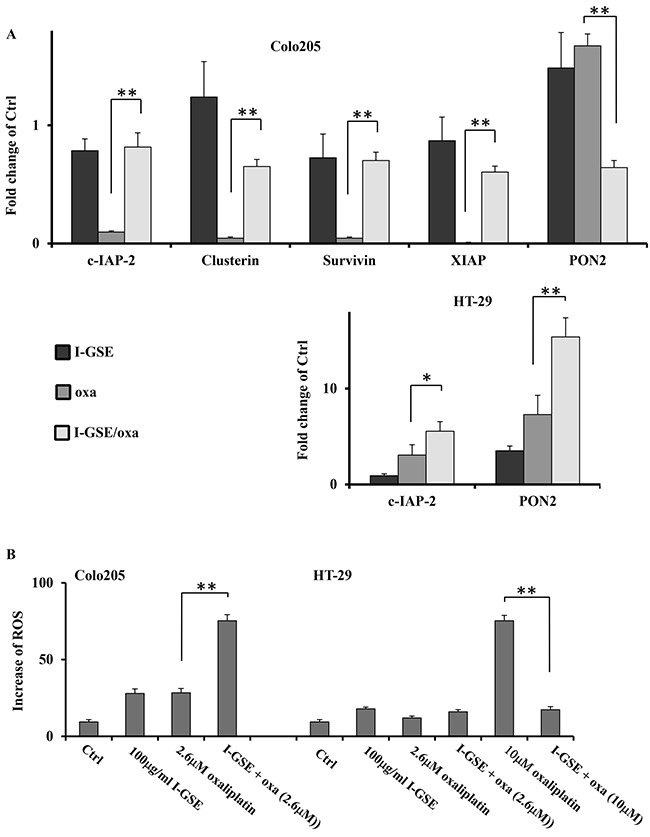
Antiapoptotic proteins and ROS generation affecting the apoptosis induced by oxaliplatin Expression quantification of protein(s) triggering anti-apoptotic response and antioxidant effect (PON2) from the densitometric analysis of the Proteome Profiler Human apoptosis array kit. The cells were treated as previously described for 3 days. Fold change of proteins expression in the treated samples respect to untreated cells ±SD, relative to two independent experiments **A.** Quantification of ROS production in HT-29 and colo205. Cells were incubated with drug(s) for 3 days and then harvested for ROS determination as described in Materials and Methods. Shown is the percentage of ROS production in treated cells compared to untreated ones ±SD, relative to three independent experiments **B.** The significance of results are: *<0.05; **< 0.001).

In agreement with previous results, in Colo205 the combination resulted in the induced expression of c-IAP-2, Clusterin, Survivin and XIAP which play a role in conveying resistance to both death receptor and mitochondrial induced apoptosis [21–23]. The analysis on the expression of these proteins, performed through the Human Apoptosis Array kit in Colo205, highlighted that I-GSE and oxaliplatin induced an opposite expression of all these proteins, however when combined the effects elicited by GSE almost completely abrogated those elicited by oxaliplatin, by shifting the balance towards the upregulation of these anti-apoptotic proteins that ultimately have the role to prevent the activation and execution mechanisms of the mitochondrial apoptosis. Basically in HT-29 the combination strongly increased the level of c-IAP-2 expression, which is involved in inhibiting the activation of caspase-3. Accordingly, despite both cellular models displayed mitochondrial damage, a very small amount of cytochrome c was released from mitochondria of HT-29 and Colo205 cells and very low level of caspase-3 activation was observed in both models (data not showed). Therefore is conceivable that the activation of the anti-apoptotic molecules exerted by GSE in Colo205 might enable the cells to cope with limited mitochondrial damage, while in HT-29 the activation of c-IAP2 can adequately block the activation of a small amount of caspase-3 reducing apoptosis (Figure 7).

Additionally, following the Human Apoptosis Array analysis, the PON2 protein resulted differently modulated by the combination of the natural extracts with oxaliplatin compared to single treatment. PON2 has been shown to prevent ROS formation impacting on both ER stress-induced pathways as well as mitochondrial proapoptotic signaling [24]. The protein analysis showed that PON2 expression was slightly induced by both GSEs and oxaliplatin and reduced when they were combined in Colo205. In HT-29, PON2 was slightly expressed in both GSE and oxaliplatin treated samples, while the extent of protein expression increased of about 10 times in the combination treated ones (Figure 7A).

Therefore to gain insights on cell stress response whether the treatments would have resulted in generation of ROS was investigated. To this purpose the oxidation-sensitive dye H2DCFDA was utilized to marker ROS accumulation within cells after treatments. Both Colo205 and HT-29 cells treated with oxaliplatinat IC_50_ or GSE alone (50μg/ml and 100μg/ml) showed a not significant and very low increase in ROS formation, while the combination induced a significant strong increase of ROS production in Colo205 and no alteration in HT-29 compared to single treatments. As expected 10μM oxaliplatin induced a pronounced increase of ROS in HT-29, however the combination with GSE overturned the situation by inhibiting ROS formation of about 70% (p<0.001) in such cells. These results are in agreement with PON2 expression, previously reported in both colon cancer models, thus is conceivable that ROS production was strictly dependent from the expression of PON2 induced by treatments (Figure 7B).

The analysis of the cellular mechanisms involved in the response to oxaliplatin when given in combination with GSEs evidenced different mechanisms of response in the chosen colon cancer models, however to complete the study it was necessary to exclude that the natural extracts could modify the oxaliplatin intracellular concentration and if so, their capability to regulate oxaliplatin import and export into cells should be investigated.

### Pt(II) accumulation

**Figure 8:**
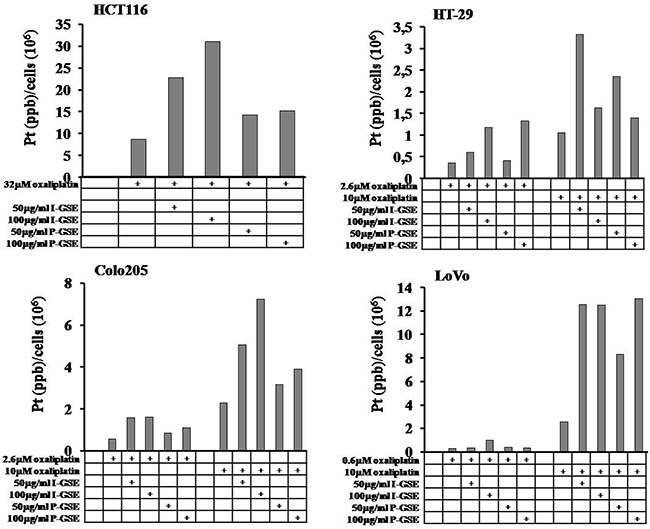
Intracellular platinum accumulation The accumulation of Pt(II) in function of the presence or absence of GSEs were measured by ICP-MS as described in Materials and Methods. Each experiment was repeated twice and changes intra-sample never exceed 10% of the value. All the differences between the pair of oxaliplatin-treated samples, in which GSEs were added or not, were statistically significant (p<0.001).

The amount of its intracellular accumulation, as platinum Pt(II), was measured by the inductively coupled plasma mass spectrometry (ICP-MS) with a Varian 820-MS ICP mass spectrometer. In each cell line, oxaliplatin was given at the IC_50_ concentration and at 10 uM concentration; in HCT116 the IC50 was 32 uM and so the drug was used only at this concentration. As expected, the higher was the oxaliplatin concentration the higher was the intracellular measured-amount of Pt(II), with an increase of about 3, 4 and 10 folds in HT-29, Colo205 and LoVo, respectively. In Figure 8 are reported the increase of Pt(II) amounts in cells after 1 day exposure to oxaliplatin in the presence or absence of GSEs. GSEs induced a strong increase of Pt(II) into cells (5-7 folds) in function of drug concentration, with the exception of HT-29 in which oxaliplatin at high dose was strongly accumulated by 50 ug/ml GSE rather than 100 ug/ml.

Curiously enough, the increased concentration of Pt(II) did not translate into increased activity of the drug as previously reported. To explain these results which seemed conflictive, the possibility that GSEs modified oxaliplatin active transport into cells was investigated.

### Transport system

**Figure 9:**
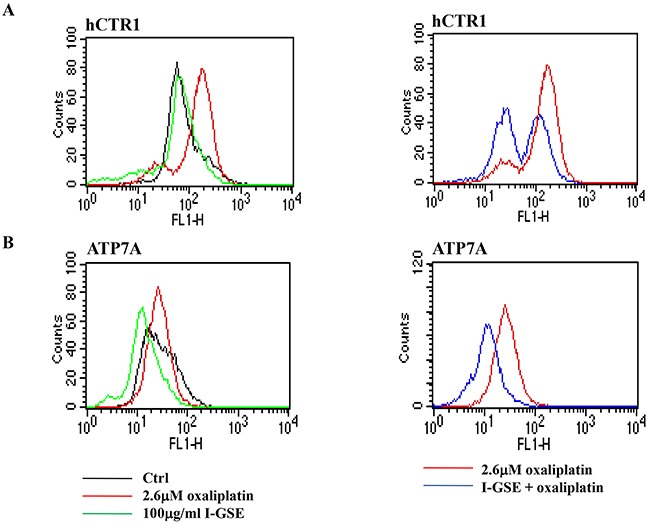
Modulation of transport system responsible for the uptake and efflux of platinum compounds Colo205 cells were treated with oxaliplatin, I-GSE and the combination for 3 days, then were fixed and analyzed for the expression of hCTR1 and ATP7A in order to verify the modulation of these transporters expression levels by FCM.

Focusing on the transport system of platinum into the cells, the capability of GSEs and oxaliplatin to modify the expression of transporters of platinum derivatives, ultimately impacting on drug uptake (through the hCTR1) and efflux (ATP7A/ATP7B) into the cells [25], was investigated. The evaluation of the expression of these transporters by FACS showed that oxaliplatin increased hCTR1 while the combination with GSEs partially reduced both hCTR1 and ATP7A (Figure 9). The two GSEs have a similar behavior that is reported in Figure 9 in which results on Lovo cells, treated with oxaliplatin and/or I-GSE, are shown. In A, is evident that GSE didn't affect the transporter hCTR1 expression conversely oxaliplatin did it; the combination of the two partially reduced the transporter expression. In B, the expression of ATP7A was reduced by GSE while oxaliplatin didn't affect it; when the two were given together, the down regulation of the transporter is maintained. The evaluations showed no modulation of ATP7B by both drugs alone or in combination (data not shown). Thus, oxaliplatin stimulated its import but didn't modify its export while both transporters were down-regulated when GSE was added.

## DISCUSSION

Recently GSEs have been showed to inhibit the growth of several cancer types [26–28] including colon cancer cells and also to increase the response to drugs, such as 5-FU, utilized for the treatment of such pathology [13]. In our study, we investigated if GSEs, from Italia e Palieri cultivars, reduced the proliferation of colon cancer cell lines and if they affected the response to oxaliplatin, by assessing the mechanisms underlying GSEs-oxaliplatin interaction in such tumors which might impact on the outcome of the oxaliplatin-based chemotherapy. Both GSEs at 100 μg/ml [29] induced a dose-dependent reduction of cell viability, however when GSEs were combined to oxaliplatin, they reduced the oxaliplatin-dependent inhibition of cell proliferation in HT-29 and Lovo cells while they did not affect the drug cytotoxicity in Colo205 and HCT116. The oxaliplatin-induced Erk1/2 inhibition plays a crucial role in the induction of the mitochondrial apoptosis by platinum derivatives [30]. Accordingly we found that in HT-29 and Lovo cells, after a strong reduction by oxaliplatin, p-Erk1/2 recovered when GSEs were added to oxaliplatin. To measure the DNA damage induced by drugs and to monitoring treatment efficacy [31], we evaluated the amount of γH2AX foci formation on DNA in both HT-29/Lovo cells and in Colo205. Interestingly, the combination induced an increase of H2AX phosphorylation in HT-29/Lovo cells and a reduction in Colo205. The increase of γH2AX is consistent with a stronger induction of the DNA damage response with concomitant arrest of cell cycle progression to allow DNA repair and genome stability restoration [31, 32], through the activation of Erk1/2 [33, 34]. The increased phosphorylation of H2AX in HT-29/Lovo cells after the addition of GSE, did not result in a further arrest of cell cycle progression but rather in the recovery of cell proliferation; therefore, we concluded that p-Erk1/2 activation in the combination, was a mechanism through which GSEs antagonized the cytotoxicity of oxaliplatin in HT-29/Lovo, ultimately determining reactivation of cell proliferation and reduction of apoptosis. In Colo205, unlike that happens in HT-29/Lovo, p-Erk1/2 was increased by oxaliplatin at late stage and not affected by the addition of GSEs, consistently with an oxaliplatin-induced apoptosis which is not dependent by mitochondria [19]. As GSEs has been reported to affect the intrinsic and/or the extrinsic pathways of apoptosis [35, 36], we evaluated the effects on: i) the activation of caspase-8 as a crucial event in death receptors apoptosis induction, ii) the truncation of BID, to assess the involvement of mitochondrial apoptosis after the death-receptors activated one, and in addition, we performed a comprehensive analysis of apoptosis by using the Human Apoptosis Array kit which allows to track simultaneously the expression/activities of several proteins involved in apoptosis. The results demonstrated that the addition of GSEs prevented the activation of the mitochondrial apoptosis and reduced the activation of the death receptors-dependent apoptosis in both Colo205 and HT-29/Lovo cells, through mechanisms that we extensively reported above. Therefore, while in HT-29 the results on apoptosis were consistent with the kinetic ones, this didn't happen in Colo205 cells.

Noteworthy in Colo205 and not in HT-29, both oxaliplatin and GSE per se, resulted in mitochondrial injury and ROS production and when combined induced a significant strong increase of ROS respect to single agent. The latter event has been reported to be required in the pro-apoptotic activation of Erk1/2 [37] and ERK activity could be directly responsible for ROS production [38]. Therefore it's conceivable that the increased ROS production in the combination supports apoptosis in Colo205, perhaps by activating Erk1/2 and basically resulting in no effect on oxaliplatin effectiveness. Conversely, in HT-29 the combination of GSE and oxaliplatin, by strongly reducing the production of ROS led to a further inhibition of the apoptotic potential of oxaliplatin. Mitochondria are the main source of ROS, however their intracellular level is determined by both their production rate and the activity of antioxidant enzymes. Interestingly while in Colo205 the addition of GSE to oxaliplatin strongly reduced the expression of PON2, in HT-29 we observed the opposite effect; i.e. the combination strongly induced the expression of PON2 compared to single treatment. Hence, GSEs in combination, by increasing PON2 expression, exerted antioxidant properties which were detrimental for the cytotoxic activity of oxaliplatin in HT-29 and Lovo cells while, by inhibiting PON2 they enhanced ROS production in Colo205 [9].

Beyond exploring the underlying mechanisms of GSE-oxaliplatin interaction impacting on cell proliferation and apoptosis, the intracellular levels of Pt(II) after administration of the GSEs were assessed, showing a puzzling result. GSEs increased the intracellular amount of the chemotherapeutic agent of about 5-7 folds compared to oxaliplatin alone, perhaps because GSEs started to inhibit the expression of the copper transporter ATP7A already after one day of treatment (data not showed) and until three days; while the expression of the CTRL1 was not inhibited after one day but after three days. Such effects on the copper transport system could account for the uptake and no efflux of oxaliplatin. However, this accumulation in cells did not seem to be followed by an increase of cytotoxic activity of oxaliplatin, in none of the colon cancer models utilized. It is not inconceivable that the increase in intracellular concentration of oxaliplatin may be responsible for an increased activity of chemotherapy or drug induced toxicity. The identification of the components of GSEs making the drug ineffective and further studies in *in vivo* models will clarify these open questions. Our ongoing study, in which GSEs will be fractionated, could allow to identify the component(s) (reported in Table 1) responsible for the reduced effectiveness of oxaliplatin, despite its high accumulation into cells, also through the analysis of a selective accumulation of the drug in the cytoplasm and/or in the nucleus. Thus, although the results reported herein did not identify which component(s) interfered with the antitumor activities of oxaliplatin in colon cancer cells, to the best of our knowledge we are the first to report an interaction of GSEs per se with oxaliplatin, according with growing evidence demonstrating that dietary supplements can mimic, intensify, or attenuate the effects of several chemotherapeutics in the treatment of colorectal cancer patients [39]. However, it should be taken into consideration that patients usually assume GSEs as crude extract and do not fractions of it, hence, our study provides preclinical evidence that GSEs like assumed by patients, might be detrimental to oxaliplatin effectiveness and therefore suggests caution in the simultaneous administration of GSEs with chemotherapeutics belonging to the class of platinum derivatives.

## MATERIALS AND METHODS

### Preparation of GSE

Grapes (*Vitisvinifera* L., cv. *Italia* and *Michele Palieri*) were sampled at harvest maturity in September 2013 from the same trial site of CREA-UTV (Turi, Southern Italy); seeds were manually removed from the grapes, washed with distilled water, drained, air-dried at 25 °C in the dark for one day, and stored at -20 °C until used. 3 g of finely ground grape seeds was accurately weighted, transferred into a 50 mL polycarbonate tube, and extracted with 30 mL methanol/water/formic acid 70:30:1 (v/v/v). The extraction was carried out in an ultrasonic bath of 130 W and 40 kHz (SONICA 2200 EP, SOLTEC, Milano, Italy) at 10 °C for 20 min. Then, the extracts were centrifuged at 4000 g and 5 °C for 3 min in an Eppendorf centrifuge 5810R (Hamburg - Germany), filtered through a 0.45 um syringe cellulose filter, and analyzed by HPLC-MS/MS. Subsequently, after completely removing the organic solvent *in vacuum* by a Speedvac Concentrator (SAVANT SPD131DDA, Thermo Scientific), the extracts were weighted and re-suspended in dimethyl sulphoxide (DMSO) to obtain 200 ug/mL stock solutions stored in aliquots at –20 °C. Further dilutions were made in medium supplemented with 10% fetal bovine serum, 2 mM glutamine, UL^-1^ penicillin and 80 uM streptomycin

### HPLC- MS/MS characterization of flvan-3-ols in GSE

The HPLC–MS system adopted in this work consisted of an HPLC 1100 (Agilent Technologies, Palo Alto, USA) equipped with a degasser (model G1379A), binary pump solvent delivery (model G1312A), thermostated column compartment (model G1316A), and a XCT-trap Plus mass detector (model G2447A, Agilent) coupled with a pneumatic nebulizer-assisted electrospray LC–MS interface. The reversed stationary phase employed was a Luna C18 (150 × 2mm i.d., particle size 3 μm, Phenomenex, USA) using a model GE1313A auto sampler (Agilent Technologies, Palo Alto, CA, USA). A pre-column, Gemini C18 5 μm (4 × 2mm i.d., Phenomenex, USA) was fitted to protect the main column. The following gradient system was used with water/formic acid (99:1, v/v) (solvent A) and acetonitrile (solvent B): 0 min, 5 % B; 10 min, 13 % B; 20 min, 15 % B; 30 min, 22 % B; 50 min 22 % B; and 55 min 5 % B; stop time to 70 min. The flow was maintained at 0.2 mL/min; sample injection was 3 μL. Negative ESI were used for ionization of molecules with capillary voltage at 4000 V and skimmer voltage at 40 V. The nebulizer pressure was 30 psi, and the nitrogen flow rate was 8 L/min. Temperature of drying gas was 350 °C. In the full-scan mode, the monitored mass range was from mass to charge ratio (*m/z*) 100 to 1200 at a scan speed of 13000 Da/s. MS/MS was performed by using helium as the collision gas at a pressure of 4.6 × 10^-6^ mbar. Collision-induced dissociation spectra were obtained with an isolation width of 4.0 *m/z* for precursor ions and a fragmentation amplitude of 0.8 V. Compounds identification was achieved by combining different information: retention time (RT), mass spectra, and the corresponding daughter MS/MS fragments were compared with those from pure standards and/or interpreted with the help of structural models already hypothesized in the literature [40, 41]. Semi-quantitation was performed using extracted ion chromatograms (EIC): for each compound, the EIC at the corresponding molecular ion was obtained and the relevant peak was integrated; subsequently, peak areas were summed with respect to the type of compound to calculate the percentage content of the different classes determined in the extracts.

### Cell lines

Human colon cancer cell lines HCT116, Colo205, LoVo and HT-29 were purchased from ATCC. Cells were routinely cultured in RPMI (HCT116 and Colo205), F-12/HAM (LoVo) or McCoy's (HT-29) medium, supplemented as above, in a humidified incubator at 37°C with an atmosphere containing 5% CO_2_. Cells were trypsinized once a week with trypsin/EDTA and the medium was changed twice a week.

### Cell proliferation assay

Determination of the concentration responsible for 50% inhibition of cell growth (IC50) was performed using the 3-[4,5-dimethylthiazol-2-yl]-2,5-diphenyltetrazoliumbromide (MTT) assay and CalcuSyn ver.1.1.4 software (Biosoft, UK) [42].

### Cell cycle analysis

After two wash steps in ice-cold PBS (pH 7.4), cells were fixed in 4.5 ml of 70% ethanol and stored at -20°C. For the analysis, the pellet was resuspended in PBS containing 1 mg/ml RNase, 0.01% NP40 and 50 ug/ml propidium iodide (PI) (Sigma). After an incubation time of 1 hour in ice, cell cycle determinations were performed using a FACScan flow cytometer (Becton Dickinson), and data were interpreted using the Cell Quest software, provided by the manufacturer.

### Cell apoptosis assay

Apoptosis detection was further investigated by the Cell Death ELISAPLUS kit (Roche Molecular Biochemicals, Milan, Italy) and by FITC Annexin V Apoptosis Detection Kit II (BD Pharmigen, USA) following manufacturers’ instructions [43].

### Cellular effectors analysis

Cells were exposed to GSEs and oxaliplatin as described in the text and protein level of the selected protein was analyzed by Western Blotting, flow cytometry (CFM) and proteome profiler array. *Western blot analysis*. Protein extracts were obtained by homogenization in RIPA buffer and treated with 1 mM phenylmethylsulfonyl fluoride (PMSF). Total proteins were measured and analyzed as described in [44]. Antibodies: the monoclonal antibody anti BID, anti Erk1/2, anti p-Erk1/2and anti-β-actin (AC-15) were provided by Cell Signalling-USA and Sigma-Aldrich. A mouse-HRP and a rabbit-HRP (Amersham Pharmacia Biotech, Upsala Sweden) were used as secondary antibody. *Flow cytometry (FCM) analysis*. Cells were harvested, washed twice in ice-cold PBS pH 7.4, fixed in 4.5 ml of 70% ethanol and stored at –20°C. Fixed cells were processed as described in our previous paper [45]. The primary antibodies were phosphor specific histone H2AX antibody (Upstate-USA), anti CTR1, anti ATP7A and anti ATP7B (Abcam, UK) and the secondary the goat anti-mouse IgG (H&L) fluorescein coniugated affinity purified secondary antibody (BD Pharmigen, USA). *Proteome profiler array*. The Proteome Profiler Human Apoptosis array kit (R&D Systems, Canada) was performed according to the manufacturer's instructions utilizing equal amounts of proteins (500 ug) from untreated and treated cells. Array images on developed X-ray film (GE Healthcare)were scanned and analyzed using Quantity One software.

### Detection of mitochondrial transmembrane potential using flow cytometry

Changes in mitochondrial membrane potential were detected by using 5,5′,6,6′-tetrachloro-1,1′,3,3′ tetraethyl benzimidazolylcarbocyanine iodide/chloride (JC-1), a cationic dye that exhibits potential-dependent accumulation in mitochondria. These changes were indicated by a fluorescence emission shift from red (590 nm) to green (525 nm), and they were analyzed by flow cytometry. Untreated and treated cells were collected, washed with phosphate-buffered saline (PBS), and analyzed on a flow cytometer.

### Determination of ROS in cells

Untreated and treated cells were washed with PBS and incubated with 10 μM dihydrodichlorofluoresceindiacetate (DCFH-DA). Intracellular DCFH-DA was deesterfied to dichlorodihydrofluorescein (DCFH) which is oxidized by ROS to produce the fluorescent compound DCFH. After incubation for 20 min at 37 °C in the dark, the cells were analyzed for fluorescence intensity by flow cytometry.

### Pt(II) accumulation

Cellular uptake of Platinum Pt(II) by colon cancer cell lines was measured by the inductively coupled plasma mass spectrometry (ICP-MS) with a Varian 820-MS ICP mass spectrometer. The cells were seeded in 60 mm tissue culture dishes at a density of ∼500000 cells and incubated at 37°C in a humidified atmosphere with 5% CO_2_. After 1 day, the culture medium was replaced with 3 ml of medium containing the tested compounds and incubated for 24 h. In particular, Oxaliplatin was given at IC_50_ concentration and at 10 uM, excepted for HCT116 where the tested concentration was of 32 uM, and GSEs were tested at 50 ug/ml and 100 ug/ml. After the incubation period, the cell monolayer was washed twice with ice-cold PBS and then digested with 2ml of HNO_3_(67%)/H_2_O_2_(30%), 1:1 (v/v), solution for 4 h at 60°C in a stove. The platinum content was determined by ICP-MS. All the experiments were performed in duplicate.

### Statistical analysis

All *in vitro* experiments were performed in triplicate, and results have been expressed as the mean ± standard deviation (SD) unless otherwise indicated. Statistical differences of *in vitro* data were assessed by the Student-Newman–Keuls test. p-values lower than 0.05 were considered significant.
